# Editorial: Oral Health and Policy for Patients With Special Health Care Needs

**DOI:** 10.3389/froh.2021.802255

**Published:** 2021-12-09

**Authors:** Duangporn Duangthip, Yasmi O. Crystal, Chun Hung Chu

**Affiliations:** ^1^Faculty of Dentistry, The University of Hong Kong, Hong Kong, Hong Kong SAR, China; ^2^College of Dentistry, New York University, New York, NY, United States

**Keywords:** oral health, children, dental caries, health policy, special health care needs, caries risk assessment

The category “people with special health care needs” refers to those with difficulties such as intellectual, physical, developmental, and medical comorbidities who require assistance. Their limitations in oral hygiene routines and access to dental care have consequently increased their risk of dental diseases, including dental caries. Previous studies have brought out the notion of dental caries in special needs patients across all age groups ranging from children to frail elders [1–3]. The severity of dental caries deteriorated with the increasing severity of intellectual disability of children [1]. Older adults with dementia exhibited a higher caries prevalence and poorer oral hygiene compared with those without dementia [3]. These patients have extensive dental treatment needs which are often inadequately met. Effective strategies including oral health policy and guidelines are required to improve oral health of people with special health care needs. Good oral health can be established by early exposure to dental services, risk assessment and check-ups, as well as a meticulous oral health prevention regimen.

The Research Topic on “*Oral Health and Policy for Patients with Special Health Care Needs”* includes a series of three articles. One study investigated the adverse behavioral effects of general anesthesia in pediatric dental patients with autism. Two reviews discussed recent practice guidelines for caries management and provided a comprehensive comparison between caries risk assessment methods.

Autism or autistic spectrum disorder (ASD) is a severe developmental disorder involving communication, socialization, and behavioral impairments. Dental care for children with autism can be a challenge for dentists. In many cases, these patients require general anesthesia (GA) when complicated or extensive treatments are performed. Tran et al. investigated the incidence of short and long term adverse behavioral effects of general anesthesia in healthy vs. moderate to severe ASD children. The authors concluded that there were significantly more short and long term adverse post-GA complications in ASD patients compared to healthy patients. The majority of adverse behavioral effects usually occurred within the first 8 and 24 h after GA. Long term adverse behavioral effects from GA possibly occur, but the chance was not high and normally not long lasting.

Tooth decay is prevalent in all age groups, including young children, elders, and those with special health care needs. A thorough understanding of the systems used for caries risk assessment is crucial. Featherstone et al. compared four caries risk assessment methods (CAMBRA, CARIOGRAM, American Dental Association, and American Association of Pediatric Dentistry caries risk assessment tools). Both the CARIOGRAM and CAMBRA are equally effective in predicting future dental caries and are both evidence-based. The authors also introduced the updated CAMBRA 123 caries risk assessment method to compare with other caries risk assessment tools in this publication. It gave equivalent results to CAMBRA and CARIOGRAM, thus providing a more practical quantitative method for caries risk assessment. The authors also reinforced the importance of identifying specific risk factors of individual patients when establishing an individualized, effective caries management plan.

Another critical review by Featherstone et al. provided updated comprehensive caries management guidelines for all age groups. These guidelines are based upon the caries risk status, results of clinical exams, and responses obtained from the caries risk assessment. The guidelines place emphasis on primary preventions such as early detection of risks factors, parental anticipatory guidance, and behavior modifications, while secondary prevention measurements such as chemical therapy along with restorative interventions can be modified according to the patient's caries risk. The implementation of the CAMBRA system of caries management based upon risk assessment can be readily implemented in dental practices and supports interprofessional collaboration by allowing professionals from different fields to evaluate oral health, educate patients, and provide appropriate referrals.

In conclusion, this Research Topic includes original and review papers emphasizing caries risk assessment and clinical practice guidelines, as well as clinical management for children with ASD using general anesthesia. These three papers in this Research Topic are beneficial for clinicians and researchers in cardiology, pediatric dentistry, dental public health, and special care dentistry. Further research is required to explore the ramifications of special needs in oral health and the techniques we need to implement to provide the utmost care for these patients.

## Author Contributions

DD, CC, and YC: conceptualization. DD: writing—original draft preparation. CC and YC: writing—review and editing. All authors have read, revised, and agreed to the published version of the manuscript.

## Conflict of Interest

The authors declare that the research was conducted in the absence of any commercial or financial relationships that could be construed as a potential conflict of interest.

## Publisher's Note

All claims expressed in this article are solely those of the authors and do not necessarily represent those of their affiliated organizations, or those of the publisher, the editors and the reviewers. Any product that may be evaluated in this article, or claim that may be made by its manufacturer, is not guaranteed or endorsed by the publisher.
